# Measuring Balance Abilities of Transtibial Amputees Using Multiattribute Utility Theory

**DOI:** 10.1155/2021/8340367

**Published:** 2021-12-21

**Authors:** Xueyi Zhang, Zhicheng Liu, Guixing Qiu

**Affiliations:** ^1^Department of Orthopaedic Surgery, Peking Union Medical College Hospital, Peking Union Medical College and Chinese Academy of Medical Sciences, Beijing 100730, China; ^2^School of Biomedical Engineering, Capital Medical University, Beijing 100069, China

## Abstract

**Background:**

Berg Balance Scale (BBS) can be considered the standard for assessment of functional balance but has a noted ceiling effect in active transtibial amputees (TTAs). Development of ceiling-free measures based on quantitative measurement techniques that is suitable for patients in any experience levels, yet sensitive enough to capture improvements in any stage of prosthetic rehabilitation, is needed. *Research Question.* Does a scoring scheme based on Multiattribute Utility (MAU) theory assess balance abilities of multileveled TTAs comparable to BBS?

**Methods:**

A case-control study including 28 participants (8 novice TTAs, 10 experienced TTAs, and 10 healthy controls) was conducted. Guided by MAU theory, a novel balance model was developed and initially validated by Spearman correlation between index-generated scores and expert assigned scores, providing preliminary evidence of validity. Floor/ceiling effects were tested, and between-group comparisons of static/dynamic balance were conducted by paired *t*-test or Wilcoxon signed-rank test depending on data distribution normality.

**Results:**

BBS score was correlated with computed balance index (*r* = 0.847, *p* < 0.001). The BBS score of novice/experienced TTAs was 39/54, and the computed balance index was 38/75. A ceiling effect of BBS (30%) was observed in the experienced TTA group, whereas no ceiling effects were found for the computed index in any combination of TTA groups. Group differences between novice and experienced TTAs were observed in center of pressure (COP) ellipse shift area, COP path length, COP average velocity, gait speed, and cadence (all *p* < 0.05). *Significance.* Evidence from first stage validation of the proposed MAU balance model indicated that the model performed well. This proposed method can monitor the progress of balance for varied experience-leveled TTAs and provide clinicians with useful information for assessing the rehabilitation training.

## 1. Introduction

Individuals with lower limb loss face gait and balance limitations. Although many factors can affect the gait and posture, balance is the only physical capacity measure shown to have a strong relationship with gait performance [1]. As part of prosthetic rehabilitation, focus is given to assessing and improving balance abilities so as to enhance self-efficacy, independence, and especially mobility safety. However, a balance assessment method that is suitable for patients in any experience level and sensitive enough to capture improvements in any stage of prosthetic rehabilitation is still needed.

Several evaluation methods have been frequently used to assess mobility and balance in persons with lower limb loss. The Berg Balance Scale (BBS) has been introduced in varied populations such as community-dwelling older adults [2], stroke survivors [3], Parkinson's patients [4], and lower limb amputees, where it has been confirmed to have excellent reliability and validity [5]. Additionally, it has been used to assess the risk of falling [6]. However, also reported were ceiling effects that may limit the utility when assessing physically active prosthesis users [7]. Timed Up and Go Test (TUG-T) [8] and One-Leg Standing Test (OLS-T) [9] are also frequently used in the clinic to assess balance or detect fall risk as performance-based measurements without the need of expensive equipment. Rather than self-report measures or tests, some authors prefer quantitative measurement techniques, such as assessments of the center of pressure (COP), plantar pressure, or spatiotemporal gait parameters in relation to posture control and gait [10, 11]. But those single-task measurements lack a detailed evaluation of functional balance.

Multiattribute Utility (MAU) theory serves to integrate a diverse set of observations or measurements into a coherent outcome with a single summary score and is increasingly used for a variety of purposes beyond economic evaluations. The MAU instruments can be a succinct indicator of health-related quality of life employed in a clinical context [12, 13]. Brennan et al. proposed MAU models for quantitative evaluation of nursing practice as opposed to relying on costly and nonreproducible global judgments by experts [14]. What the MAU needs is a set of attributes, scales to measure each attribute, and the weight that designates the relative contribution of each individual attribute to the overall performance.

For a balance model, attributes derived from varied quantitative measurements (static/dynamic balance) that are mostly used in functional balance evaluations would allow diverse important parameters to be entered into one analytical model. This paper presents a novel MAU approach to create a balance model and demonstrates its use of scoring algorithms to generate an index for evaluating global balance. Additionally, a preliminary validation of the balance model against the BBS scores assigned by clinicians is conducted.

### 1.1. Subjects

A sample of unilateral transtibial amputees (TTAs) was recruited for this Institutional Review Board-approved study. Participants were included from two groups, which were experienced TTAs (from outpatients) and novice TTAs (from inpatients undergoing their first-time prosthetic fitting). A sample of healthy nonamputee adults was included as the controls. For the experienced TTAs, inclusion criteria were an activity classification of K-level 2 to K-level 4, with experience in prostheses use for more than one year. Individuals needed to have no existing skin damage or limb pain. Individuals with abnormal sensory function (e.g., visual impairment and vestibular organ disease) or with neurological disorders that affect balance (such as stroke) were excluded from participation. Informed written consent was obtained prior to the data collection. The target sample size was 30 (10/group).

### 1.2. Protocol Design

#### 1.2.1. Berg Balance Scale

The BBS was proposed by Katherine Berg in 1989 [15]. It can measure static and dynamic balance ability among individuals by observing COP shifting when completing various functional activities in sitting and standing positions. The degree of success in achieving each task (14 tasks in total) is given a score of 0, 1, 2, 3, or 4, and the final measure is the sum of all scores. The lowest possible total score is 0, and the highest is 56. A score lower than 40 indicates a risk of falling.

#### 1.2.2. Static Balance Attributes Extraction

Participants were asked to stand upright on the Zebris PDM-S measurement platform (Zebris Medical, Munich, Germany). The sampling frequency was 100 Hz. Participants were instructed to keep balance while standing, and their feet should be parallel to avoid plantar pressure changes caused by COP displacement. Standing balance tests were done with shoes on, since there are few instances of barefoot standing or walking with the prosthesis in daily life. Three trials per subject were conducted with each lasting 15 seconds. The values were averaged for the final results.

A shift of COP is an indirect measure of postural sway and also a measure of a TTA's ability to maintain balance. The static balance attributes include COP ellipse shifts area (mm^2^), COP path length (mm), and COP average velocity (mm/s). Taking into account that the novice unilateral TTAs could hardly load bodyweight symmetrically on both legs at first, the percentage ratio of plantar pressure on affected versus sound side (%) was measured. Additionally, the percentage ratio of left versus right plantar pressure in the healthy participants (%) was computed for comparison.

#### 1.2.3. Dynamic Balance Attributes Extraction

Participants were asked to walk at a self-selected speed along a 30-meter corridor. Gait data were collected simultaneously by a G-walk sensor (BTS Bioengineering, Milan, Italy), a wearable sensor that has been widely used in research involving lower limb amputees [16, 17] or healthy people [18]. The wireless triaxial accelerometer device was fixed to the fifth lumbar vertebrae with an ergonomic belt, which allowed subjects' unimpeded walking. The data was transmitted to a computer at a 100 Hz sampling frequency through Bluetooth. In recent literature [18] on the reliability and concurrent validity of G-walk, it was reported that the G-walk sensor is reliable for all measured spatiotemporal parameters, with excellent concurrent validity for gait speed, cadence, stride length, and poor to moderate validity for single/double support time and swing/stance duration.

Through this test, ten gait parameters including gait speed (m/s), cadence (step/min), percentage of stance phase (%), swing phase (%), double support time (%), and single support time (%) on each side were obtained. In this study, six of ten variables (gait speed, cadence, stance phase L%, stance phase R%, double support L%, and double support R%) that are most strongly related to dynamic balance were selected and extracted as dynamic balance attributes.

#### 1.2.4. Procedures

Standing balance tests were performed twice for novice TTAs: immediately after prosthetic fitting (T0) and on discharge day (T1) (range of T0 to T1 was 18 to 32 days). Between T0 and T1, in the course of the initial management of the amputees as inpatients in a rehabilitation unit, they were given training that included standing in parallel bars, carrying weight, shifting COP, walking inside/outside the parallel bars. This rehabilitation training is routine after prosthesis fitting for a new amputee. Walking tests were performed only once at T1, as participants in this group were mostly unable to walk at T0. For the experienced TTA or healthy subjects, none of the rehabilitation training was conducted. Standing balance and walking tests were scheduled only once in a random order with ten minutes' rest in between to avoid fatigue. BBS scores were recorded as clinical evaluation of balance abilities for all the participants.

### 1.3. Model Building

MAU theory provides the theoretical basis to translate the assessment of functional balance tests into a multidimensional evaluation scheme. The theory also supports the development of an index where a weighted sum reflects the extent to which a balance model achieves functional balance ideals. Classical MAU theory approaches rely on a complex elicitation process to build the attribute hierarchy by experts, estimate the subjective valuation for each attribute scale, establish weights, and confirm the computational function [19]. These subjective analytical demands have discouraged the use of MAU theory as an objective measurement device.

In our context, nine quantitatively accessed gait parameters mostly reflecting the static or dynamic balance abilities were employed as attributes. Entities vary in the degree or amount of attribute in order to represent the nature and distribution. Attributes are described by numerical or phrase-anchored scales, in which the scale values reflect the degree or amount of the attribute possessed by the entity. The values on these scales are referred to as single-attribute utilities, and they are specific for each entity. In the case of the MAU model used in this study, the utility of specific attributes (such as speed) was used as the measurement outcomes.

To determine the utility of the attributes, normalization was achieved by averaging the value of each attribute from the lowest to highest score by using the calculation described by Edwards and Newman [20]:

(1) $$\begin{array}{c} L_{\text{a}}=\frac{L_{\text{a}}-L_{\min}}{L_{\max}-L_{\min}}, \end{array}$$

where *L*_a_ is the actual location value of a particular attribute, *L*_max_ is the maximum value, and *L*_min_ is the minimum value of *L*_a_.

Then, the attributes of COP ellipse shift area, COP path length, and COP average velocity as negative indicators were transformed as needed to be positive indicators. This is to reflect that a larger value in the aforementioned attributes denotes a lower balance capacity.

Weights were assigned to each attribute to identify its contribution to the final results, as they are unlikely to be of equal importance. It is necessary to normalize the weights so that the weights of all attributes sum up to 1:

(2) $$\begin{array}{c} 0<W_{\text{i}}<1=1,2,\cdots N,\\ \overset{N}{\underset{i=1}{\sum }}W_{i}=1. \end{array}$$

To determine the weight for each attribute, a more objective scheme—coefficient of variation (CV)—was employed:

(3) $$\begin{array}{c} C_{i}=\frac{\sigma_{i}}{\mu_{i}}, \end{array}$$

where *C*_*i*_ is the CV of an attribute, *σ*_*i*_ is the standard deviation, and *μ*_*i*_ is the mean of the attribute. The rule of the calculation is that the greater the data differentiate, the greater the attribute weight. In this way, the final index will be well able to discern differences in the comprehensive evaluation system, while avoiding the ceiling effects. The final normalized weight of each attribute was computed as follows, and results are shown in Table 1:

(4) $$\begin{array}{c} W_{I}=\frac{C_{i}}{\sum_{i=1}^{N}C_{i}}. \end{array}$$

To choose a functional form for the MAU model, MAU theory suggests that when two or more attributes can independently have a large impact on overall benefit, a multiplicative or multilinear model is appropriate [21]. The functional form in this study was chosen as follows:

(5) $$\begin{array}{c} \text{BI}={\left(\overset{N}{\underset{i=1}{\sum }}W_{i}\times A_{i}^{k}\right)}^{1/k}, \end{array}$$

where BI is the balance index, *N* is the total number of attributes, *W*_*i*_ is the weight of attribute *i*, *A*_*i*_ is the utility of attribute *i*, and *k* equals 1 in this study.

### 1.4. Validation and Statistical Analysis

The test of the ability of an MAU model to mimic human judgment aims to demonstrate that the balance ability scores computed by the index match those assigned through the clinician's appraisal. Upon the finding that data distribution was nonnormal, a nonparametrical Spearman's correlation coefficient was used to assess the correlation between the computed balance index and the exact BBS score to validate the balance model. The strength of association was defined as weak (<0.5), moderate (0.5–0.8), or strong (>0.8). Flooring/ceiling effects were calculated as the percentage of participants who achieved the minimum or maximum possible BBS score. Flooring or ceiling effects of 20% or greater were considered clinically significant [22]. To identify differences between the novice TTAs (at T1) and experienced TTAs, various statistical tests were performed. Normality of parameters of static and dynamic balance was assessed based on the Shapiro-Wilk test. Paired *t*-tests were applied if data was normally distributed; otherwise, Wilcoxon signed-rank tests were applied. All statistical tests were carried out with IBM SPSS version 24. The level of significance was set at *α* < 0.05.

## 2. Result

Thirty participants were recruited, of which 28 completed the protocol (8 novice TTAs, 10 experienced TTAs, and 10 healthy controls) and were included in the analysis (Table 2). For the static balance tests, the parameters of COP ellipse shift area (*p* < 0.001), COP path length (*p* < 0.05), and COP average velocity (*p* < 0.05) tested at T1 were significantly larger in novice TTAs than the respective values in experienced TTAs (Table 3). The plantar pressure on the prosthesis side for novice TTAs increased from T0 to T1; however, it did not reach the levels seen in experienced TTAs. Across all parameters, there were significant differences at T1 between novice TTAs and experienced TTAs. As for the dynamic balance, gait speed (*p* < 0.001) and cadence (*p* < 0.001), respectively, were significantly lower for novice TTAs than experienced TTAs (Table 4).

Comparing to the healthy individuals, COP ellipse shift area, COP path length, COP average velocity, gait speed, and cadence were all significantly different between novice and experienced TTAs. The parameters plantar pressure, stance phase, and double support time of healthy individuals were used to determine normative range for comparison. They were analyzed as a ratio of left/right side in healthy individuals and of affected/sound side in TTAs.

The BBS scores and computed balance index are shown in Table 5. Spearman correlation between the BBS score and the computed balance index was 0.847 (*p* < 0.001) when all the TTAs were included. For the novice TTAs, it was 0.929 (*p* = 0.038), higher than the combined groups, while it was 0.004 (*p* = 0.817) when only experienced TTAs were included.

As shown in Table 6, a ceiling effect of BBS was observed for few of the prosthesis users (3/18). In the group of novice TTAs, no ceiling effects were exhibited at all (0/8). However, for the experienced TTAs, the BBS scores were clustered at the top of the scale, with several subjects reaching the maximum score of 56 resulting in ceiling effects of 30% (3/10). By comparison, no ceiling effect was observed for the computed balance index in any combination of prosthesis user groups. Healthy subjects as the control group all reached the perfect BBS score.

## 3. Discussion

Our balance models arose from the structural and contextual dimensions of functional balance tests and were applicable to every balance dimension. Evidence from our first stage validation of the balance model indicates that the model performed well. By extension, with intangible, theoretical formulations that support the translation of human perception into numerical scaling strategies provide the necessary formalization of clinician judgment into practical research tools. The nature of attributes included in our model parallels those proposed in other studies of functional balance evaluation with COP or gait assessment instruments in terms of scope, topical theme, and diversity [23, 24]. Han et al. [23] proposed that by tracking the path of COP using the F-scan insole system during stance phase, the balance and pattern of progression can be determined. Huijben et al. [24] suggested that lower walking speed of older adults results in lower gait quality, which underlies the differences that can contribute to falling risks. Considering that these key variables reflect one or several aspects of gait quality or balance abilities, our method of devising a composite score based on more detailed evaluation of functional balance appears to be suitable to monitor the progress of rehabilitation in extension of the applications of aforesaid researches.

The floor/ceiling effects of BBS in people with transtibial amputation were also evaluated in this study. The BBS performance by the experienced TTAs (BBS score, mean 54 (range 50-56)) and its difference to the novice TTAs' scores was comparable to that of other populations of prosthesis users, including transtibial or transfemoral prosthesis users (53 (49-55) vs. 52 (49-54)) [7], users or nonusers of ambulatory aids (52 (47-56) vs. 41 (34-49)) [25], and fear or not of falling (49 (47-52) vs. 53 (50-55)) [7]. Our results suggest that slight ceiling effects exist in experienced TTAs, and 30% of these participants achieved the maximum score of BBS. Similar ceiling effects have been reported for several other pathological conditions that cause unsteadiness [22, 26, 27]. No ceiling effect was observed when the sample size only included novice TTAs. This suggests that TTAs with low balance ability may be more suitably assessed by BBS, and without incurring the ceiling effects. Similarly, Azuma et al. [25] reported no ceiling effects in transfemoral amputees, with most participants over age 60 having low BBS scores, even though younger participants had close to perfect BBS scores. Therefore, to address this problem, our proposed method could be a complementary balance assessment tool for active prosthesis users.

After amputation, novice TTAs needed a certain amount of time to resume standing and walking. More importantly, they are facing challenges in mobility safety at the early stage of resuming their regular life after hospital discharge. Before our study, no BBS scores of hospitalized novice TTAs have been reported. Our finding of BBS scores of 39 (35-46) points out a considerable risk of falling in this population. The results in Table 3 detail how especially three parameters relative to COP were, respectively, significantly larger for the novice than the experienced TTAs, indicating a greater risk of balance-related problems and falling. As for the dynamic balance, recent work [24] suggested that lower gait speed is indicative of lower gait quality in older adults, which can be extrapolated to aged prosthesis users. This was confirmed by our results in Table 4, showing that the novice TTAs had a significantly lower gait speed and cadence than the experienced TTAs. Although the healthy subjects were more stable while standing and faster while waking, the results of experienced TTAs may be interpreted as a benchmark of balance ability after returning to the society. To monitor the recovery over time, our method appears suitable and sensitive enough to capture improvements of functional balance for a broad population of prosthesis users.

## 4. Limitations

One limitation of this research is the small sample size, as the recruited participants were split into three groups. However, the sample size is comparable to many studies in prosthetics and orthotics. A future larger-scale study has been motivated by the here presented preliminary findings.

It is possible that the core attributes of the balance model may change over time. In this initial validation, the performance of the MAU model was sufficient to support further testing. A field study to determine the performance of the balance model to match new prosthetists evaluating new patients could provide evidence of generalizability.

## 5. Conclusion

In this paper, we proposed a novel method to provide useful evaluation of functional balance in various experience levels of TTA by quantitative measurement techniques. Guided by MAU theory, nine factors became elements in a computational index that when summed, assigns a score to a given patient reflecting the extent to which that patient's balance ability approximates able-bodied levels. Spearman correlation between the index-generated scores and the expert assigned scores provided evidence supporting the preliminary validation of the balance model.

## Figures and Tables

**Table 1:** Weight assigned to each attribute.

Attributes	Weight
COP ellipse shift area	0.18
COP path length	0.18
COP average velocity	0.18
Gait speed	0.08
Cadence	0.05
Stance phase A%	0.02
Stance phase S%	0.03
Double support A%	0.17
Double support S%	0.11

**Table 2:** Participant demographics.

Subject	Sample	Age (years)	Height (m)	Weight (kg)	Time since amputation (months)
Novice TTA	8	41.5 ± 10.9	1.64 ± 0.09	72.2 ± 12.6	7 ± 2 (5-10)
Experienced TTA	10	40.6 ± 11.2	1.74 ± 0.08	76.5 ± 8.5	57 ± 30 (25-112)
Healthy controls	10	32.8 ± 6.5	1.74 ± 0.06	66.5 ± 9.4	——

Novice TTA denoting new prosthesis users with initial hospitalized; outpatient denoting the experienced prosthesis users. Results are shown as mean ± SD. Time since amputation is shown as mean ± SD (range).

**Table 3:** Static functional balance tests.

	Novice TTA		Experienced TTA	Healthy controls
	T0	T1		
COP ellipse shifts area (mm^2^)	1240.33 ± 124.29	676.78 ± 122.31	253.33 ± 136.87^∗∗^	138.47 ± 52.96
COP path length (mm)	583.55 ± 63.87	357.50 ± 60.58	135.94 ± 55.22^∗^	89.87 ± 23.43
COP average velocity (mm/s)	39.33 ± 4.28	24.28 ± 4.32	9.34 ± 3.55^∗^	6.43 ± 1.62
Plantar pressure A (%)	26.93 ± 0.66	40.45 ± 1.17	47.50 ± 7.85^∗^	——
Plantar pressure S (%)	73.17 ± 0.66	59.56 ± 1.17	54.30 ± 6.93^∗^	——
Plantar pressure L (%)	——	——	——	49.40 ± 1.06
Plantar pressure R (%)	——	——	——	50.27 ± 0.98

Paired *t*-test was applied if the data was normally distributed by the Shapiro-Wilk test. Otherwise, nonparametric. The Wilcoxon signed-rank test was applied. Healthy controls are shown as normal range. ^∗^*p* < 0.05 and ^∗∗^*p* < 0.001.

**Table 4:** Dynamic functional balance tests.

	Novice TTA	Experienced TTA	Healthy controls
Gait speed (m/s)	0.77 ± 0.07	1.17 ± 0.16^∗∗^	1.33 ± 0.11
Cadence (step/min)	73.50 ± 6.02	99.64 ± 7.29^∗∗^	123.04 ± 5.22
Stance phase A (%)	61.77 ± 4.04	58.57 ± 3.01	——
Stance phase S (%)	61.61 ± 4.14	62.19 ± 5.76	——
Double support A (%)	7.50 ± 1.11	11.05 ± 6.07	——
Double support S (%)	14.23 ± 3.54	12.79 ± 5.56	——
Stance phase L (%)	——	——	60.33 ± 1.57
Stance phase R (%)	——	——	60.7 ± 1.53
Double support L (%)	——	——	10.01 ± 1.16
Double support R (%)	——	——	9.97 ± 1.13

Paired *t*-test was applied if the data was normally distributed by the Shapiro-Wilk test. Otherwise, nonparametric. The Wilcoxon signed-rank test was applied. Healthy controls are shown as normal range. ^∗^*p* < 0.05 and ^∗∗^*p* < 0.001.

**Table 5:** Berg Balance Scale score and computed balance index.

	Berg Balance Scale score	Computed balance index
Novice TTA	39 (35, 46)	38 (22, 47)
Experienced TTA	54 (50, 56)	75 (49, 96)
Healthy controls	56 (56, 56)	——

Results are shown as mean (range).

**Table 6:** Flooring and ceiling effects of Berg Balance Scale scores.

	Flooring effects	Ceiling effects
Novice TTA	0 (0)	0 (0)
Experienced TTA	0 (0)	3 (30%)^∗^
All prosthetic subjects	0 (0)	3 (16.7%)

All prosthetic subjects were combined novice and experienced TTA groups. Results are shown as number (%) of subjects who reached minimum or maximum possible BBS score. ^∗^Significant effect was over 20%.

## Data Availability

The data sets are available from the corresponding author.
